# Integrated knowledge translation (iKT) in preclinical research: A scoping review protocol

**DOI:** 10.1371/journal.pone.0337202

**Published:** 2025-11-20

**Authors:** Georgia Black, Reena Besa, Daniel Blumberger, Heather Brooks, Graham Collingridge, John Georgiou, Evelyn K. Lambe, Clement Ma, Bernadette Mdawar, Tarek K. Rajji, Sanjeev Sockalingam, Cara Sullivan, Quincy Vaz, Zhengbang Yao, Branka Agic

**Affiliations:** 1 Community Health Sciences, University of Calgary, Calgary, Alberta, Canada; 2 Centre for Addiction and Mental Health, Toronto, Ontario, Canada; 3 Department of Psychiatry, Temerty Faculty of Medicine, University of Toronto, Toronto, Ontario, Canada; 4 Temerty Centre for Therapeutic Brain Intervention, Centre for Addiction and Mental Health, Toronto, Ontario, Canada; 5 Department of Physiology, Temerty Faculty of Medicine, University of Toronto, Toronto, Ontario, Canada; 6 Lunenfeld-Tanenbaum Research Institute, Toronto, Ontario, Canada; 7 Tanz Centre for Research in Neurodegenerative Diseases, University of Toronto, Toronto, Ontario, Canada; 8 Department of Obstetrics and Gynaecology, Temerty Faculty of Medicine, University of Toronto, Toronto, Ontario, Canada; 9 Clinical Public Health Division, Dalla Lana School of Public Health, University of Toronto, Toronto, Ontario, Canada; 10 UT Southwestern Medical Center, Dallas, Texas, United States of America; 11 The Wilson Centre, University Health Network, Toronto, Ontario, Canada; 12 Lived Experience Consultant, Centre for Addiction and Mental Health, Toronto, Ontario, Canada; Public Library of Science, UNITED KINGDOM OF GREAT BRITAIN AND NORTHERN IRELAND

## Abstract

**Introduction:**

Integrated knowledge translation (iKT) is a collaborative research approach that emphasizes the meaningful and active participation of knowledge users throughout the research process. Evidence suggests that integrated knowledge translation has the potential to increase the relevance, applicability, and use of research findings. This approach has been increasingly utilized in health research in recent years. However, the extent to which it has been applied in preclinical research and its effectiveness are unknown. To address this gap, we will conduct a scoping review to map the current use, potential benefits, and challenges of iKT in preclinical research.

**Methods:**

Guided by a modified Arksey and O’Malley’s scoping review framework, we will systematically search reference lists and key research databases including Medline, Embase, PsycINFO, Cochrane CENTRAL, Cochrane Database of Systematic Reviews, and Web of Science. Peer-reviewed articles written or translated in English that focus on iKT or approaches that align with iKT within the context of preclinical research will be included. This review will be conducted as part of the **I**mproving **N**europlasticity through **S**paced **P**refrontal **i**ntermittent-Theta-Beta-Stimulation **RE**finement in **D**epression (**INSPiRE-D)** project, which features preclinical research from mouse models to human work (Grant number CAMH File No.22-060). The project’s multidisciplinary team and knowledge user advisory committee will be consulted at key points throughout the scoping review process. A person with lived experience co-chairs the project advisory committee, co-authored this manuscript, and will be routinely included in the decision-making process of the scoping review.

## Introduction

### Knowledge translation: Bridging the ‘Know Do’ Gap

Knowledge translation (KT) is a dynamic process that aims to increase the impact and uptake of research findings through the “synthesis, dissemination, exchange and ethically sound application of knowledge.” [1]. KT is grounded in meaningful interactions between researchers and *knowledge users*: members of groups who are likely to use research results to inform their decisions such as clinicians, policy makers, patients and families, funders, and others for which the research holds significance [1–3].

KT is critically important in bridging a well-documented ‘know-do’ gap between knowledge gained through research and its application in policy and practice so that individuals and communities can fully and timely benefit from research discoveries [4,5]. As such, significant resources have been allocated to advancing KT strategies over the past two decades, including more recently the use of an integrated knowledge translation (iKT) approach [1,6].

### Integrated knowledge translation

Unlike traditional end-of-grant KT where research results are typically shared through outputs generated at the final stage of a research study, iKT is a collaborative approach to knowledge translation where researchers partner with knowledge users throughout the entire lifecycle of a research project from conceptualization to dissemination [7–10]. Originally developed within the Canadian health research context, iKT has risen to prominence in recent years due to its promotion by the Canadian Institutes of Health Research (CIHR) federal funding agency as a means of encouraging the collaborative creation and uptake of research knowledge [6,11].

While iKT shares many of the core values of collaborative research approaches commonly employed in engaged scholarship, it has some distinguishing features [6,12]. Compared to other collaborative approaches, iKT explicitly emphasizes the meaningful inclusion of knowledge users throughout the research process from inception to dissemination including identifying research priorities, developing research questions, interpreting findings, and advancing knowledge translation [1,6,13]. In addition, iKT focuses on achieving specific ends related to improving health outcomes, services, and systems [6]. Evidence suggests that iKT may enhance the relevance, usability, and impact of research findings [8,10], in addition to raising knowledge users’ understanding of each other’s worlds and building stronger relationships that could lead to future research collaboration [7,8]. In recognition of its potential efficacy and endorsement by CIHR, iKT has been increasingly utilized in health research involving human participants in recent years [8]. However, the extent to which an iKT approach has been applied in preclinical research, i.e., research prior to involving human participants, is currently unknown.

### iKT in preclinical research

The difficulties of translating knowledge from preclinical to clinical (‘bench to bedside’) contexts and beyond are well-documented [14], with an entire field of translational research dedicated to bridging this gap. More recently though, researchers have explored whether the engagement of knowledge users in preclinical research may enhance knowledge translation [15–18].

This current discussion is exemplified in two recent scoping reviews exploring patient engagement and public involvement in preclinical research [15,16]. While not focusing on iKT specifically, these reviews highlight the apparent gap in the literature on the inclusion of knowledge users, in particular the public and patients, in preclinical research. Both reviews found that patients and members of the public are often not involved throughout key phases of preclinical research. For example, Fox et al. [15] found that while there were examples of patient engagement in preclinical studies, these were somewhat limited with patients less likely to be involved during the data collection and data analysis phases. Equally, Carroll et al. [16] found that there were no examples in the literature of patient involvement at the commissioning or implementation stages of preclinical research. However, the reviews also identified potential benefits of engaging patients and members of the public in preclinical research including opportunities to align preclinical research priorities with the needs of patients, recognize the real-life implications of the research findings, and improve knowledge dissemination [15,16]. With its focus on engaging a broad range of knowledge users throughout the entire research process and emphasis on increasing the uptake of research findings, iKT may be an effective approach in preclinical research. However, how iKT has been applied in a preclinical context or the extent to which knowledge users have been involved is unclear.

To address this gap, we will conduct a scoping review of peer-reviewed literature to map the current landscape around iKT in preclinical research. The findings of this review may also serve to provide further insights on the methodological application of iKT in the preclinical context compared with other collaborative research approaches summarized in existing literature reviews [15,16]. The scoping review is part of the **I**mproving **N**europlasticity through **S**paced **P**refrontal **i**ntermittent-Theta-Beta-Stimulation **RE**finement in **D**epression (**INSPiRE-D)** project. This three-year research project aims to bridge preclinical to first-in-human studies. The overarching goal is to optimize the delivery of transcranial magnetic stimulation that will potentiate synapses in the prefrontal cortex in people with major depression. The preclinical focus is to identify the most efficient theta-burst pattern and to pinpoint underlying mechanisms that could be targeted in the future by pharmacological interventions to augment transcranial magnetic stimulation clinical effects in humans. Since this bench-to-bedside collaboration is aimed at closing the gap between preclinical and clinical research, it gives a unique perspective into the incorporation of iKT into different types of research.

### Aim, objectives, research questions

The review will be guided by the overarching research question: **What is known in the peer-reviewed literature about the use of iKT in preclinical research?** This will be addressed through the following three specific objectives:

1) To map the current peer-reviewed literature on the use of iKT within preclinical research contexts
2) To identify benefits and challenges to employing iKT approaches in preclinical research documented in the peer-reviewed literature
3) To inform the development of a model of bench-to-bedside collaboration aiming to close the gap between preclinical and clinical research and beyond.

## Methods and analysis

This work will be guided by the scoping review methodology outlined by Arksey & O’Malley [19] and later refined by Levac et al. [20] and Peters et al. [21]. This methodology typically involves (1) identifying the research question, (2) developing and applying a search strategy to obtain relevant papers, (3) screening and selecting search results according to inclusion and exclusion criteria, (4) extracting and charting data in line with the review objectives, (5) analyzing and reporting the results, and (6) consulting relevant stakeholders. Step 6 is often positioned as an end-of-project phase intended to improve the knowledge translation of scoping review findings. However, we have modified this approach to include knowledge users as a necessary first step. The Advisory Committee for the iKT component of the INSPiRE-D project is composed of key knowledge users such as People with Lived Experience (PWLE), psychiatrists, basic scientists, clinicians and trainees. The co-chair of this committee and a co-author of this manuscript (CS) is a caregiver to a PWLE and is routinely included in the decision-making process of the scoping review. The research team will consult with the INSPiRE-D iKT Advisory Committee at key points throughout the review with the aim of increasing the relevance, usability, and dissemination of the review findings. In line with best practice, our review will follow the Preferred Reporting Items for Systematic Reviews and Meta-Analyses extension for Scoping Reviews (PRISMA-ScR) [22]. Covidence, a systematic review management software, will be used to manage key review tasks such as importing citations, screening titles and abstracts, reviewing full texts, and extracting data [23].

1 **Identifying the research question**

Under the overarching research question, “What is known in the peer-reviewed literature about the use of iKT in preclinical research?”, further sub-questions that will be explored include: “What benefits and challenges of implementing iKT in preclinical settings have been identified in the literature?” In this review, the **Population** of interest is *knowledge users*, defined by CIHR [1] as “individuals who are likely to be able to use research results to make informed decisions about health policies, programs, and/or practices.” The central **Concept** is *iKT,* which CIHR [1] describes as “an approach to doing research that applies the principles of knowledge translation to the entire research process, wherein knowledge users collaborate as equal partners with the explicit aim of increasing the relevance, applicability, and uptake of research findings.” The **Context** of this review is *preclinical research*, which encompasses research that occurs prior to any human studies. This definition includes in vitro, ex vivo, in vivo, and in silico (i.e., cell system, biological sample, living animal or computer model) experiments in laboratory settings [15].

2 **Identifying relevant studies**

A comprehensive search strategy will be developed with support from a research librarian. No search limits on geographical location or date will be applied. After piloting, refining, and finalizing, the research librarian will apply the strategy to search Medline, Embase, PsycINFO, Cochrane CENTRAL, Cochrane Database of Systematic Reviews, and Web of Science. To ensure that we capture as many relevant papers as possible, we will also hand-search reference lists of review articles for relevant papers. Search results will be uploaded and de-duplicated in Covidence.

3 **Study selection**

The inclusion and exclusion criteria (detailed in Table 1 below) have been iteratively developed with members of the study team and discussed with the INSPiRE-D iKT Advisory Committee. Broadly speaking, articles will only be included if they are available in English and focus on iKT and/or knowledge user engagement approaches that align with an iKT approach within the context of preclinical research. Unpublished or non-peer-reviewed literature will be excluded. A random sample of the results from the search strategy will be used to modify the specificity of the inclusion and exclusion criteria. Two levels of screening will follow: a title and abstract review to identify articles meeting the inclusion criteria and a full-text review of those passing the first screen. Two independent reviewers will perform each level of review and compare and discuss results. Any disagreements will be resolved by a third reviewer (BA), the study’s Principal Investigator, with expertise in knowledge translation methods and strategies.

**Table 1:** Inclusion and exclusion criteria.

	Inclusion Criteria	Exclusion Criteria
**Type of Literature**	Peer-reviewed literature including original research articles, commentary, thematic papers, editorials, research letters, reviews.	Unpublished or non-peer-reviewed literature incl. conference proceedings, white papers, reports, briefings, meeting minutes, news/magazine articles)
**Language**	Written or translated in English only.	Any other language with no English translation.
**Date limit**	N/A	N/A
**Geographical limit**	N/A	N/A
**Topic**	Literature that focuses on iKT or knowledge user engagement approaches aligned with iKT in preclinical research, guided by the following definitions: • Population: ‘Knowledge Users’ defined as “individuals who are likely to be able to use research results to make informed decisions about health policies, programs, and/or practices.” [1] • Concept: ‘iKT’ defined as “an approach to doing research that applies the principles of knowledge translation to the entire research process, wherein knowledge users collaborate as equal partners with the explicit aim of increasing the relevance, applicability, and uptake of research findings.” [1] • Context: ‘Preclinical Research’ which includes in vitro, ex vivo, in vivo, and in silico (i.e., cell system, biological sample, living animal or computer model) experiments in laboratory settings [15].	• Literature that focuses on approaches that cannot be categorized as iKT (e.g., patient engagement, involvement, or consultation not focusing on increasing the uptake of research findings; end of grant knowledge translation). **AND/OR** • Focuses on non-preclinical settings (e.g., human studies, clinical trials, psychosocial studies).

4 **Charting the data**

The core research team will initially develop a draft extraction sheet which they will pilot using a sample of five to ten included articles. The draft extraction sheet will be shared with the full research team and presented at the INSPiRE-D iKT Advisory committee meeting for feedback. Based on the findings from this piloting and consultation phase, the extraction sheet will be refined. Examples of potential data categories include author(s), year of publication, location and area of research, and characteristics of knowledge users. For knowledge translation, we will extract data on the goals and rationale for iKT, evaluation of iKT activities, iKT outputs, benefits and challenges, enablers and limitations, and recommendations for improving KT practices. Data extracted will include both discrete categories (e.g., yes/no, numerical information) as well as open responses. Once the final extraction sheet has been developed, it will be uploaded to Covidence where two reviewers will independently complete the extraction template for each included article, with conflicts resolved by a third reviewer. Team members completing extractions will meet on a regular basis to identify any further data themes that emerge during this phase that should also be captured in the review.

5 **Collating, summarizing and reporting the results**

Once the extraction phase is complete, the data will be exported into a Microsoft Excel sheet where it will be prepared for analysis. The analysis will be exploratory in nature and we will employ a descriptive qualitative approach, aligning with the primary goal of this scoping review to map the literature and identify knowledge gaps. Descriptive statistics will be generated to provide an overview of the key data categories. Excerpts of text will be analyzed through discussions among the three extractors to identify common themes, knowledge gaps, and opportunities for future research. The findings will be discussed with the iKT Advisory Committee to validate or interpret the findings.

The research team will work with the iKT Advisory Committee to develop a robust knowledge translation strategy for the scoping review results and broader INSPIRE-D project. This will include creating tailored knowledge products for specific audiences (e.g., peer-reviewed publications, presentations, research snapshots, infographics, etc.), in addition to using the findings from this review to inform the other components of the INSPiRE-D project.

6 **Consultation**

The INSPiRE-D iKT Advisory Committee comprising knowledge users such as clinicians, PWLE, trainees, and basic and clinician scientists will be engaged from the beginning of the scoping review process and during each phase as the study progresses. Regular updates on the review process will be presented and discussed at the INSPiRE-D project team and Advisory Committee meetings. They will provide input regarding the search strategy, interpreting and summarizing the results, as well as the development of knowledge translation products and strategies.

## Patient and public involvement statement

The co-chair of the INSPiRE-D iKT Advisory Committee, who is a caregiver to a PWLE, is a named co-author of the manuscript. She (CS) will be involved with the development of the search and extraction strategy, the interpretation and translation of the study findings, and the writing and review of subsequent manuscripts. Two additional PWLE will be consulted as members of the Advisory Committee throughout the review process. All PWLE will receive honoraria for their contribution. Authorship recognition will align with the International Committee of Medical Journal Editor Guidelines [24].

## Ethics and dissemination

Research ethics board approval is not required for this scoping review. The conduct of this work will align with ethical standards for developing partnerships with patients and researchers, as outlined by CIHR [25]. The findings from the scoping review will be published in an open-access, peer-reviewed journal and presented at local, national, and international conferences. The research team will work with the knowledge user advisory committee to develop and disseminate knowledge products tailored to specific audiences.

## Study timeline and progress

The scoping review process has been ongoing since April 2023, with multiple revisions to the initial search terms to yield relevant articles. We are currently at Step 5 of the Arksey & O’Malley [19] methodology, where we are actively collating and summarizing findings from the scoping review in consultation with the INSPIRE-D research team and iKT Advisory Committee. The review is expected to be completed by July 2025.

## Discussion

iKT is an approach that engages knowledge users and researchers in mutually beneficial research, which has been increasingly utilized in health research involving human participants in recent years. In the clinical research literature, iKT has been associated with the potential to increase the relevance, applicability, and uptake of research findings [8,10,13]. At the same time, challenges have been documented, including balancing diverse stakeholder priorities, sustaining engagement, and navigating power dynamics among knowledge users [7,12]. Yet, the extent to which iKT is used in preclinical research is unknown, nor do we know its potential benefits and challenges in this preclinical context.

This scoping review will generate evidence on the application of iKT in preclinical research and provide a basis for exploring the unique considerations that may arise in this setting, where the iKT pathway and *knowledge users* landscape differ from those in clinical research. For instance, preclinical iKT may present additional barriers to engagement for knowledge users because of their likely lack of familiarity with preclinical concepts, terminology, and the potential downstream impact of such research. Bridging this potential gap may require a greater investment in education on preclinical terminology and methods to ensure meaningful integration, a theme this review will help to explore.

### Strengths and limitations

The results of this scoping review will add new knowledge regarding the current application of iKT in preclinical research.

Knowledge users including scientists with a background in preclinical and clinical research, clinicians, and patients and family members with lived experience will be engaged throughout the review and dissemination process.

Due to our lack of language resources, this review will be restricted to English-language peer-reviewed publications, which may limit the generalizability of the review findings.

Preprints, trial registries, and other forms of grey literature were excluded from this review. As iKT is a collaborative approach that spans the full research lifecycle, protocols for studies that are still in progress provide only intended plans for iKT and do not yield data on its implementation, outcomes, or challenges. However, it is acknowledged that this exclusion may limit the capture of emerging evidence and early insights into iKT practices that have not yet reached the stage of peer-reviewed publication.

## Supporting information

S1 Appendix Sample search strategy. (DOCX)

S1 Checklist PRISMA-ScR checklist. (PDF)

S2 Checklist PRISMA-P checklist. (DOCX)
