# Supplementary material for: Integrated knowledge translation (iKT) in preclinical research: A scoping review protocol
Source: PLoS One. 2025 Nov 20;20(11):e0337202. doi: 10.1371/journal.pone.0337202 (PMC12633911; doi:10.1371/journal.pone.0337202)
Supplement: S1 Appendix — (DOCX) [file pone.0337202.s001.docx]

Draft MEDLINE Search – Ovid Interface

(integrat* adj2 knowledg* adj2 translat*).ab,kf,hw,ti.

ikt.ab,kf,hw,ti.

biomedical translation/

knowledge translation/

patient participation/ and (research or knowledge).ab,kw,hw,ti.

Patient Advocacy/ and (research or knowledge).ab,kw,hw,ti.

Consumer Advocacy/ and (research or knowledge).ab,kw,hw,ti.

(((client or clients or consumer or consumers or "knowledge user" or "knowledge users" or patient or patients or public or "service user" or "service users" or stakeholder or stakeholders) adj1 (activation or advocacy or advocat* or empower* or engage* or involv* or orientation or participat* or represent*)) and (research or (knowledge adj1 translation*))).ab,kf,hw,ti. /freq=2

Research co-production.ab,kf,hw,ti.

(knowledge adj (translation or mobilization or transfer)).ab,kf,hw,ti.

Translational Science, Biomedical/

Translational Research, Biomedical/

exp *animals/ and (*research/ or *biomedical research/)

((animal or animals) adj3 (research or knowledge)).ab,kf,hw,ti.

((basic or fundamental or lab-based or translation*) adj1 (research or science)).ab,kf,hw,ti.

"basic and clinical science".ab,hw,kf,ti. /freq=2

bench research.ab,kf,hw,ti.

(laborator* adj3 (research or knowledge)).ab,kf,hw,ti.

((pre-clinic* or preclinic*) adj3 (experiment or research* or trial* or study or studies)).ab,kf,hw,ti.

(translation* adj2 research).ab,kf,hw,ti.

or/1-2 [ikt]

(or/3-7) and (or/11-13) [knowledge translation (KT)/patients participation Subject Headings (SH) AND translational science/research/preclinic (SH)]

(or/8-10) adj5 (or/14-20) [(KT/patients participation) ADJ# (translational science/research/preclinic) Keywords]

21 or (or/22-23) [ikt OR patient engagement/preclinic]

(research or (knowledge adj translation*)).ab,kf,kw,ti. /freq=2

24 and 25
